# Identification of Specific miRNA Signature in Paired Sera and Tissue Samples of Indian Women with Triple Negative Breast Cancer

**DOI:** 10.1371/journal.pone.0158946

**Published:** 2016-07-12

**Authors:** Seema Thakur, Rajesh K. Grover, Sanjay Gupta, Ajay K. Yadav, Bhudev C. Das

**Affiliations:** 1 Dr. B.R. Ambedker Centre for Biomedical Research, University of Delhi, New Delhi, India; 2 Delhi State Cancer Institute, Delhi, India; 3 Guru Teg Bahadur Hospital, Delhi, India; 4 Stem Cell & Cancer Research Lab, Amity Institute of Molecular Medicine & stem cell Research (AIMMSCR), Amity University, Uttar Pradesh, Sector 125, Noida-201313, India; University of South Alabama Mitchell Cancer Institute, UNITED STATES

## Abstract

Of several subtypes of breast cancer, triple negative breast cancer (TNBC) is a highly aggressive tumor that lacks expression of hormone receptors for estrogen, progesterone and human epidermal growth factor receptor 2 and shows a worst prognosis. The small noncoding RNAs (miRNAs) considered as master regulator of gene expression play a key role in cancer initiation, progression and drug resistance and have emerged as attractive molecular biomarkers for diagnosis, prognosis and treatment targets in cancer. We have done expression profiling of selected miRNAs in paired serum and tissue samples of TNBC patients and corresponding cell lines and compared with that of other subtypes, in order to identify novel serum miRNA biomarkers for early detection and progression of TNBC. A total of 85 paired tumor tissues and sera with an equal number of adjacent normal tissue margins and normal sera from age matched healthy women including tissue and sera samples from 15 benign fibroadenomas were employed for the study. We report for the first time an extremely high prevalence (73.9%) of TNBC in premenopausal women below 35 years of age and a significant altered expression of a panel of three specific oncogenic miRNAs- miR-21, miR-221, miR-210, and three tumor suppressor miRNAs- miR-195, miR-145 and Let-7a in both tissues and corresponding sera of TNBC patients when compared with triple positive breast cancer (TPBC) patients. While miR-21, miR-221 and miR-210 showed significant over-expression, miR-195 and miR-145 were downregulated and well correlated with various clinicopathological and demographic risk factors, tumor grade, clinical stage and hormone receptor status. Interestingly, despite being a known tumor suppressor, Let-7a showed a significant overexpression in TNBCs. It is suggested that this panel of six miRNA signature may serve as a minimally invasive biomarker for an early detection of TNBC patients.

## Introduction

Breast cancer is the most common cancer and a leading cause of cancer-related mortality in women worldwide. In India about 1, 44,937 women were diagnosed with breast cancer in 2012 and approximately 90,000 deaths occur every year [1, 2]. Of several subtypes of breast cancer, the most aggressive form is characterised by absence of estrogen receptor (ER), progesterone receptor (PR) and human epidermal growth receptor 2 (HER2) and designated as triple negative breast cancer (TNBC) [3]. The drug resistance and recurrence rate of TNBCs is very high and it accounts for 10–24% of all breast cancers [4–6]. Most alarmingly and unlike in US and Europe, TNBC is highly prevalent in Indian women at premenopausal stage (<35 year) when they are in the prime time of their reproductive life. Although mammography, ultrasound and Fine Needle Aspiration Cytology (FNAC) are the current standard diagnostic tools and various other diagnostic and prognostic biomarkers have been reported, there has been not much reduction in the incidence and mortality of triple-negative breast cancer [7]. This is because TNBCs lack both diagnostic as well as therapeutic targets which is a major unmet public health need. Therefore, there is a need to develop a novel, non-invasive diagnostic approach to improve reliable detection and therapy of TNBCs. Recently, alteration in expression of non-coding small RNA molecules (microRNAs) has been considered as the most reliable diagnostic biomarker in various cancers including breast cancer.

miRNAs (microRNAs) are small ~19–25 nucleotides long non-coding RNA sequences which play a pivotal role in regulating gene expression by inhibiting translation and/or triggering degradation of coding mRNAs by binding to complementary sequences found in the 3′ untranslated region (UTR). miRs are important players in regulation of various biological processes including cell differentiation, proliferation and apoptosis. Several lines of evidence indicate that altered expression of miRNAs is present in various types of human cancers, which can serve as a potential biomarker for the diagnosis and progression/prognosis of various cancers. Emerging data shows that specific miRNAs may play a potential regulatory role in carcinogenic process either by functioning as tumor suppressor genes or oncogenes. Several studies have also demonstrated that different types of cancer at different developmental stages display unique expression profile of different microRNAs [8–10]. Thus use of specific microRNA (miR) expression signatures may serve as diagnostic and/or prognostic biomarker of a tumor [11, 12].

microRNAs are often released into the blood circulation by apoptotic and necrotic cells as well as by active secretion as exosomes which remain stable in blood. Exosomes are important mediators of intercellular communication and play a pivotal role in tumor progression and metastasis. Blood serum being the primary biological specimen representing the largest profile of human proteome, profiling of miRs in serum could provide more valuable information on the pathogenesis of cancer [13, 14]. The dysregulated miRs expression level appears to reflect distinct molecular phenotype of breast cancer [15]. Therefore, circulating miRs have been used not only as early diagnostic and/or prognostic biomarkers but also for classification of breast cancer. We have therefore selected three oncogenic and three tumor suppressor miRNAs which are most frequently reported to be involved in breast carcinogenesis [16, 17]. We demonstrate here that a panel of six specific circulating miRNAs may serve as a potential non-invasive biomarker which correlates very well with ER/PR/EGFR-2 status and clinicopathological parameters for an early and reliable diagnosis of TNBC patients, majority of whom are premenopausal in India.

## Materials and Methods

### Patients and samples

A total of 85 paired samples (n = 85) of blood serum and breast tumor biopsy along with adjacent normal tissue margins from untreated breast cancer patients and serum samples from 85 age matched normal healthy women accompanied with the patients and 15 benign breast adenoma tissue biopsies and paired sera along with adjacent normal tissue and sera from 15 age matched normal women, were collected from Delhi State Cancer Institute (DSCI) and Guru Teg Bahadur (GTB) Hospital, Dilshad Garden, Delhi. All cases were histopathologically confirmed and written informed consent was taken before collecting the samples. The study was approved by the Institutional Ethics Committee of ACBR, University of Delhi, DSCI and GTB Hospital, Delhi. The freshly operated breast tumor tissue samples were collected in sterile vials containing RNA Later and were stored at -80°C until further analysis. All histopathologically confirmed untreated cases of breast carcinoma were included in this study. Breast cancer cases with other diseases or malignancy were excluded from the study. The patients were recruited randomly as they were presented in the cancer OPD of the hospital.

### Blood collection and serum preparation

5 ml of peripheral venous blood was collected from each of the breast cancer patients and from healthy volunteers in a serum separator tube without any anticoagulant. Patient’s blood was collected prior to surgery or any definitive therapy. Collected blood was allowed to stand at room temperature for a minimum of 30 min and centrifuged at 1,200 g at 4°C for 15 min and isolated serum was aliquoted (500 μl) into 1.5 ml eppendorf tubes, which were stored at −80°C until RNA extraction.

### Cell Culture

Human breast cancer cell lines MCF-7 and MDA-MB-231 obtained from American type culture collection (ATCC, USA) were grown in Dulbecco’s modified Eagle medium (DMEM) containing 10% heat-inactivated fetal bovine serum (FBS), 2 mM L-glutamine and penicillin and streptomycin (100 IU/mL and 100 μg/mL, respectively) at 37°C with 5% CO_2_. Normal breast epithelial cell line, MCF-10A cells as control was grown in DMEM-F12 (Life Technologies) supplemented with 5% horse serum, 0.5 μg/ml hydrocortisone (Sigma), 10 μg/ml insulin (Sigma), 20 ng/ml epidermal growth factor (Sigma) and antibiotics.

### RNA extraction

#### i.) From breast tissue samples

The frozen tissues were homogenized in lysis buffer. Total RNA was extracted from tumor and normal tissues of the breast by mirVana^™^miRNA isolation kit (Ambion, USA) using manufacturer’s protocol. Nucleic acid concentration and purity were determined by using Nanodrop ND-1000 (Thermo Scientific, USA).

#### ii.) From serum samples

The frozen sera were thawed and 250 μl was transferred into an eppendorf tube. Isolation of total RNA from serum samples of cancer patients and normal controls was simultaneously carried out using miRNeasy kit, (Qiagen) according to manufacturer’s protocol. RNA concentration and its purity was determined by Nanodrop ND-1000.

#### iii.) From cultured cells

The total RNA was extracted from breast cancer and normal breast epithelial cell lines using mirVana^™^ miRNA isolation kit (Ambion, USA) according to manufacturer’s protocol for tissue RNA extraction.

### Quantitative real-time PCR

cDNA was synthesized from 20 ng RNA extracted from serum, tissue and cell lines using specific miRNA RT primers of Taq-Man MicroRNA Reverse Transcription kit (Applied Biosystems, USA) and manufacturer’s protocol. Real-Time PCR was performed separately for tissue, sera and cell lines with their corresponding normal controls using the TaqMan universal matermix kit (Applied Biosystems, USA) and its protocol. Reverse transcription as well as Real-Time PCR for miRNA expression analysis was carried out using primers for hsa-miR-21, hsa-miR-221, hsa-miR-210, hsa-miR-195, hsa-miR-145 and hsa-miR-Let-7a and SnRNA U6 was used as a reference control. The threshold cycle data were determined using the default threshold settings. Real time PCR was performed in a 10 μl reaction volume containing 2 μl of RT product (c-DNA), 1x Taqman universal master mix (5μl), 1 μl of Taqman miRNA assay and 2 μl H_2_O to get a final volume. We have always used same concentration on cDNA for all miRNA analysis in order to maintain same efficiency. The reaction profile was 10 min at 95°C, followed by 45 cycles of 95°C for 15s and 60°C for 1 min. All experiments were run in triplicate, along with a negative and a positive control. The fold change of miRNA expression for each breast tumor sample in relation to normal control was calculated based on the threshold cycle (CT) value using the following formula: Relative Quantification (RQ) = 2^−ΔΔCT.^

### Statistical analysis

Statistical analysis to detect the difference in the miRNA expression level between breast cancer and normal tissue or serum sample and cell lines was done using the One -Way ANOVA test followed by Bonferroni post hoc test for significant difference. This was used to compare the mean response. Data were presented as means±SE of three or more independent experiments. Independent t-test was carried out and the difference was considered statistically significant when P< 0.05. The correlation between miRNA expression and clinicopathological features was analyzed by Pearson's correlation coefficient (r). Receiver operating characteristic (ROC) curves were constructed and the area under the curve (AUC) was calculated to assess the ability of each miRNA to differentiate between TNBC and controls, by computing sensitivity and specificity for each possible cut-off point of the individual miRNA. This was performed univariately for each individual and comparative miRNAs, and combined ROC of miRNAs were computed by multiple logistic regression. All the data were analysed using GraphPad Prism version 5 and SPSS v 16 software.

## Results

Out of a total of 85 breast cancer cases recruited randomly for the present study, twenty three (27%) were triple negative breast cancer with a mean age of 36.27± 10.27 and a majority of them (69.56%) were in early age group below 35 years and premenopausal (73.91%) (see Table 1). But majority (95.16%) of other subtypes of breast cancer were in age group above 35 years with a mean age of 51.15±11.22. Obviously a majority (74%) of women with TNBC were in premenopausal stage (p = 0.004) (Table 1). Younger age, Pre- menopausal state, early menarche, smoking, high mitotic activity, higher expression of Ki67 and Lymph node negativity were found to be significantly associated with an increased risk for TNBC (Table 1). In contrast, older age, post- menopausal stage, late menarche, lymph node positivity and non- vegetarianism, were mostly associated with an increased prevalence of other subtypes of breast cancer (Table 1).

**Table 1:** Clinicoepidemiological characteristics of TNBC and other subtypes of breast cancer patients.

Triple Negative breast cancer (n = 23)					Other breast cancer (n = 62)	
S. NO	Clinicopathological variables	Category	Number of cases n (%)	P value	Number of cases n (%)	P value
**1.**	**Age Range & (Mean ±SD)**	(21–70)	23(36.27±10.27)		62(51.72±9.27)	
**2.**	**Age Group distribution**	<35	16(69.56)	0.020*	3(4.83)	0.0001*
		≥35	7(30.43)		59(95.16)	
**3.**	**Menopausal Status**	Pre- menopausal	17(73.91)	0.004*	20(32.25)	0.0025*
		Post- menopausal	6(26.08)		42(67.74)	
**4.**	**Menarche Status**	<13	17(73.91)	0.004*	42(67.74)	0.0026*
		≥13	6(26.08)		20(32.25)	
**5.**	**Oral contraceptive use**	Ever	15(65.21)	0.062	36(58.06)	0.1021
		Never	8(34.78)		26(41.93)	
**6.**	**Smoking Status**	Yes	16(69.56)	0.020*	24(38.70)	0.0375*
		NO	7(30.43)		38(61.29)	
**7.**	**Food habits**	Vegetarian	9(39.13)	0.142	17(27.41)	0.0001*
		Non- Vegetarian	14(60.86)		45(72.58)	
**8.**	**Religion Status**	Hindu	10(43.47)	0.263	34(54.83)	0.0008*
		Muslim	13(56.52)		28(45.16)	
**9.**	**Mitotic activity Status**	High	18(78.26)	0.0005*	52(83.87)	0.0001*
		Low	5(21.73)		10(16.12)	
**10.**	**Ki67 (cell proliferation marker)**	Positive	18(78.26)	0.0005*	50(80.64)	0.0001*
		Negative	5(21.73)		12(19.35)	
**11.**	**Histopathological grade**	I+II	15(65.21)	0.06	46(74.19)	0.0001*
		III	8(34.78)		16(25.80)	
**12.**	**Clinical Staging**	Stage I+II	12 (52.17)	0.165	35(56.45)	0.1548
		Stage III+IV	11(47.82)		27(43.54)	
**13.**	**Lymph node status**	Positive	7(30.43)	0.020*	36(58.06)	0.1021*
		Negative	16(69.56)		26(41.93)	
**14.**	**Body mass index(BMI) (kg/m**^**2**^**)**	Obesity (≥34)	14(60.86)	0.142	25(40.32)	0.0637
		Non-obesity (<25)	9(39.13)		37(59.67)	
**15.**	**Hereditary status**	Hereditary	2 (8.6)	0.0001*	4(6.45)	0.0001*
		Sporadic	21 (91.30)		58(93.54)	
**16.**	**BRCA1 Status**	Mutated	13(56.52)	0.281	7(11.29)	0.0001*
		Non-mutated	10(43.47)		55(88.70)	
**17.**	**BRCA2 Status**	Mutated	4(17.39)	0.0001*	15(24.19)	0.0001*
		Non-mutated	19(82.60)		47(75.80)	

* Significant.

**Fig 1:**
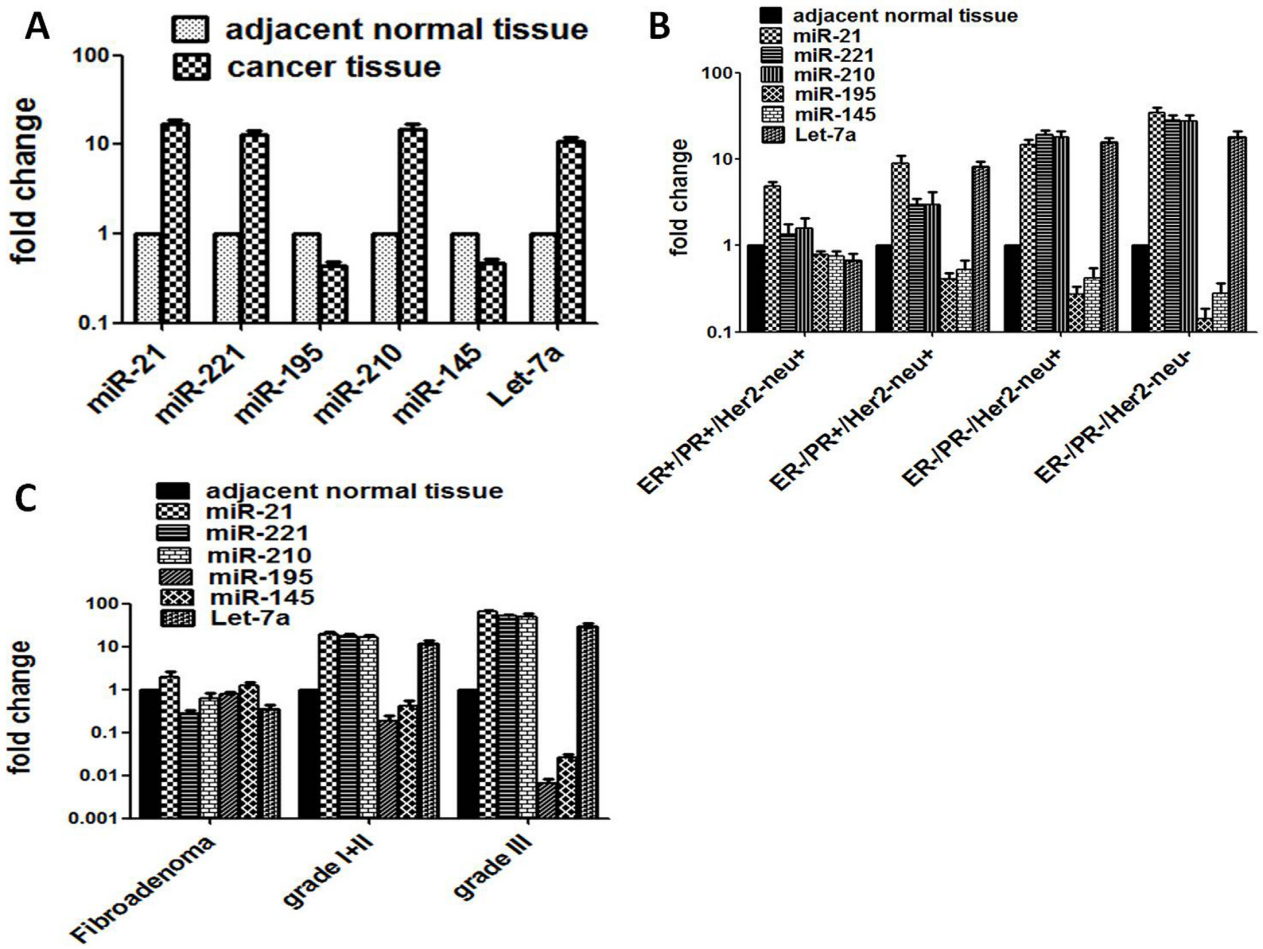
The expression level of six specific miRNAs in tissue specimens from TNBC and other subtypes of breast cancer, benign breast cancer and controls. (A) The expression level of miR-21, miR-221, miR-210, miR-195, miR-145 and Let-7a in total breast tumour and adjacent normal tissues. (B) Expression profiles of miR-21, miR-221, miR-210, miR-195, miR-145 and Let-7a of TNBC patients and other hormonal subtypes, triple positive breast cancer or TPBC (ER+/PR+/Her2neu+), single negative breast cancer or SNBC (ER-/PR+/Her2neu+) and double negative breast cancer or DNBC (ER-/PR-/Her2neu+) along with adjacent normal tissue controls. (C) Expression level of miR-21, miR-221, miR-210, miR-195, miR-145 and Let-7a in fibroadenoma (benign breast tissue) and different tumor grades from breast cancer patients and normal tissues.

**Fig 2:**
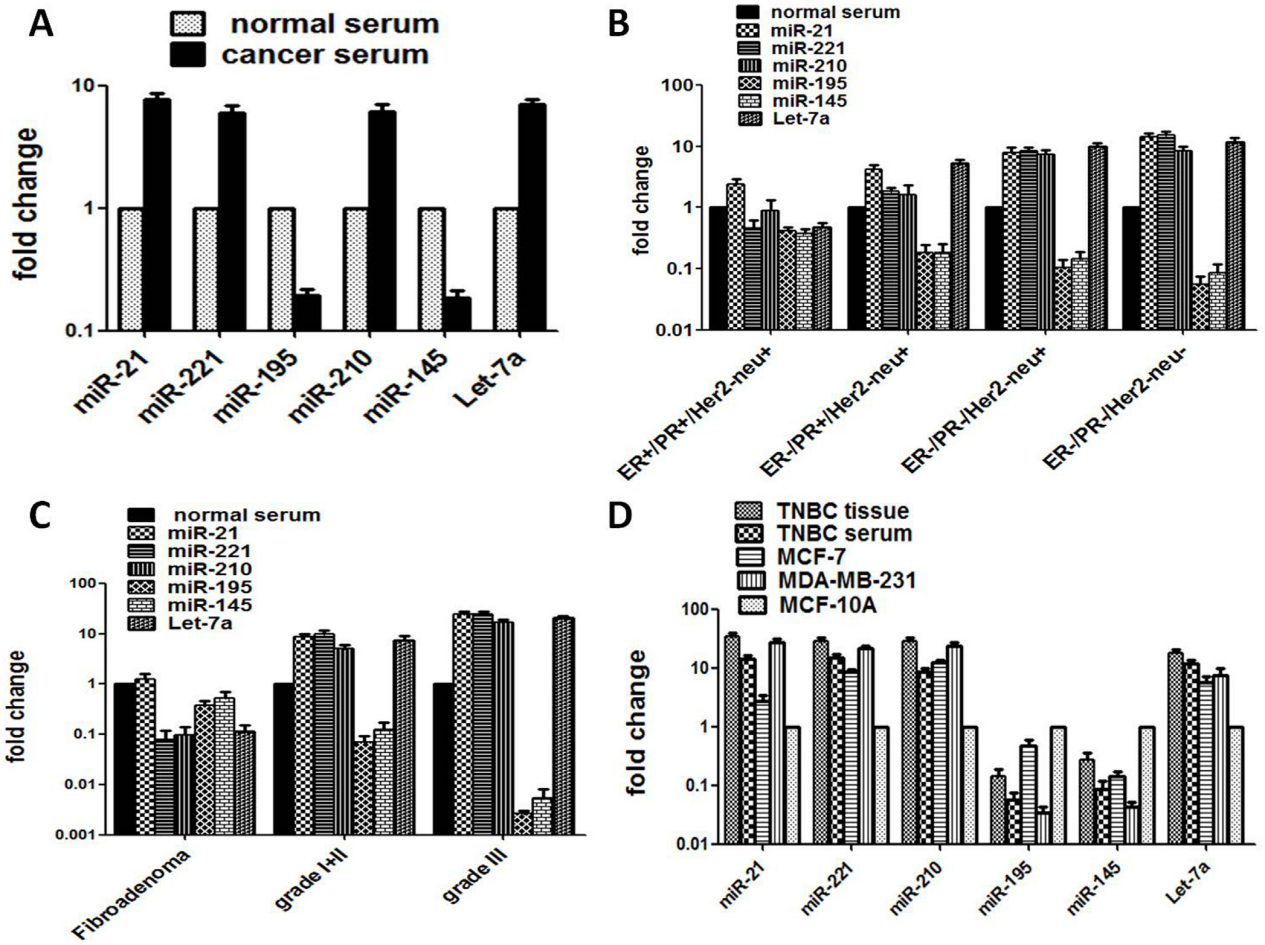
The expression level of six specific miRNAs in serum specimens from TNBC and other subtypes of breast cancer, benign breast cancer and controls. (A) Expression level of selected miRNAs (miR-21, miR-221, miR-210, miR-195, miR-145 and Let-7a) in total serum samples from breast cancer patients and healthy individuals. (B) Expression profiles of miR-21, miR-221, miR-210, miR-195, miR-145 and Let-7a in TNBC patients and other hormonal subtypes (ER+/PR+/Her2neu+), (ER-/PR+/Her2neu+) and (ER-/PR-/Her2neu+) in sera from breast cancer patients along with healthy individuals. (C) Expression level of miR-21, miR-221, miR-210, miR-195, miR-145 and Let-7a in sera of fibroadenoma and different tumor grades of breast cancer and healthy controls. (D) Comparison of **e**xpression level of miR-21, miR-221, miR-210, miR-195, miR-145 and Let-7a in TNBC tissue, sera and cell lines (MCF-7 and MDA-MB-231) with normal mammary epithelial cell line MCF-10A.

Since an altered expression level of six selected microRNAs, miR-21, miR-221, miR-210, miR-145, miR-195 and Let-7a is known to be frequently involved in breast carcinogenesis, these have been examined in paired TNBC tissue and serum samples in comparison to that of adjacent normal tissue margins and normal serum. We also included tissue biopsy and sera from benign fibroadenoma for comparison and correlated their tissue/sera expression profile of these six miRNAs with that of TNBC patients and breast cancer cell lines, MDA-MB-231 (TNBC, ER-/PR-/Her2neu-) and MCF-7 (DPBC, ER+/PR+/Her2neu-) along with normal breast epithelial cell line (MCF-10A). The results were correlated with clinicopathological parameters as well as epidemiological risk factors including hormone receptor status, tumor grades and clinical stages (Table 1 and S1 Table). The level of expression of oncogenic miR-21, miR-221, miR-210 including the tumor suppressor miR Let-7a were found to be consistently up-regulated in TNBC tissues as well as in their corresponding sera and cancer cell lines, MDA-MB-231, MCF-7, whereas miR-145 and miR-195 were regularly downregulated (p<0.001) (Table 2a and 2b, Figs 1A, 2A and 2D).

**Table 2:** The level of miRNA expression in breast cancer tissue and paired sera in different hormonal subtype of breast cancer.

**(a) In breast cancer tissue (n = 85)**												
**Hormonal status**												
**miRNA Types**	**TPBC** (n = 21)			**SNBC** (n = 20)			**DNBC** (n = 21)			**TNBC** (n = 23)		
	**FC±S.E**	**Regulation**	**P value**	**FC±S.E**	**Regulation**	**P value**	**FC±S.E**	**Regulation**	**P value**	**FC±S.E**	**Regulation**	**P value**
miR-21	4.90±0.51	up	0.001*	8.85±2.05	up	0.0001*	14.59±2.09	up	0.0001*	34.641±5.22	up	0.0001*
miR-221	1.33±0.41	up	0.417	2.97±0.53	up	0.001*	19.05±2.56	up	0.001*	28.35±4.04	up	0.001*
miR-210	1.57±0.50	up	0.255	2.99±1.11	up	0.82	18.00±2.86	up	0.0001*	27.82±4.52	up	0.001*
miR-195	0.78±0.08	down	0.007*	0.41±0.07	down	0.001*	0.27±0.06	down	0.001*	0.14±0.04	down	0.001*
miR-145	0.76±0.09	down	0.008*	0.52±0.15	down	0.001*	0.41±0.12	down	0.001*	0.27±0.08	down	0.001*
Let-7a	0.67±0.13	down	0.001*	8.25±1.07	up	0.001*	15.61±1.76	up	0.001*	17.90±2.93	up	0.001*
**(b) In breast cancer sera (n = 85)**												
**miRNA Types**	**TPBC** (n = 21)			**SNBC** (n = 20)			**DNBC** (n = 21)			**TNBC** (n = 23)		
	**FC±S.E**	**Regulation**	**P value**	**FC±S.E**	**Regulation**	**P value**	**FC±S.E**	**Regulation**	**P value**	**FC±S.E**	**Regulation**	**P value**
miR-21	2.42±0.52	up	0.005*	4.17±0.77	up	0.001*	7.84±1.77	up	0.001*	13.99±2.11	up	0.0001*
miR-221	0.45±0.17	down	0.003*	1.81±27	up	0.006*	8.24±1.36	up	0.001*	14.97±2.24	up	0.001*
miR-210	0.88±0.42	down	0.41	1.59±0.74	up	0.868	7.30±1.27	up	0.0001*	8.39±1.34	up	0.0001*
miR-195	0.41±0.05	down	0.001*	0.18±0.06	down	0.001*	0.10±0.03	down	0.001*	0.05±0.01	down	0.001*
miR-145	0.37±0.07	down	0.001*	0.18±0.06	down	0.001*	0.14±0.04	down	0.001*	0.08±0.03	down	0.001*
Let-7a	0.47±0.08	down	0.001*	5.22±0.77	up	0.001*	9.80±1.38	up	0.001*	11.84±1.99	up	0.001*

**Abbreviations:** TPBC: Triple Positive Breast Cancer; SNBC: Single Negative Breast Cancer; DNBC: Double Negative Breast Cancer; TNBC: Triple Negative Breast Cancer; FC: Fold change; S.E: Standard Error mean;

* Significant.

### Increased expression of specific microRNAs in tumor tissue of triple negative breast cancer

We analyzed the expression pattern of a set of six selected microRNAs- miR-21, miR-221, miR-210, miR-145, miR-195 and Let-7a in four subtypes of breast cancer tissues including adjacent normal tissues. The hormone receptor based four subtypes of breast cancer considered throughout the study were (i) ER+/PR+/Her2neu+ (triple positive breast cancer, TPBC); (ii) ER-/PR+/Her2neu+ (single negative breast cancer, SNBC); (iii) ER-/PR-/Her2neu+(double negative breast cancer, DNBC) and (iv) ER-/PR-/Her2neu- (triple negative breast cancer, TNBC). Out of six microRNAs screened, four of them, miR-21; miR-221; miR-210 including the tumor suppressor miR Let-7a were consistently upregulated whereas two miR-145 and miR-195 were regularly downregulated in TNBC tissue as compared to that of adjacent normal tissue (Table 2a). The fold change expression levels of miR-21, miR-221, miR-210 and Let-7a were 34.64, 28.35, 27.82 and 17.90 respectively which were significantly different from those of normal breast tissues but also from all other breast cancer (BC) subtypes (see Table 2a; Fig 1B). Expression of these six miRs changed as a function of hormone receptor positivity/negativity and/or severity of breast cancer with TNBC being the highest. Out of 6 miRNAs, exclusively, miR-21 was found to be highly sensitive as it consistently showed a significantly (p < 0.0001) much higher level of expression in all breast cancer subtypes. When we compared TNBC with TPBC patients it exhibited significantly a highest fold change expression of miR-21 (34.64 vs 4.90; p < 0.001), followed by miR-221 (28.35 vs 1.33; p < 0.001), miR-210 (27.82 vs 1.57; p < 0.001) and Let-7a (17.90 vs 0.67; p< 0.001) (Table 2a). In comparison to other breast cancer subtypes, DNBC, SNBC and TNBC patients also showed significantly a higher expression of miR-21, miR-221, miR-210 and Let-7a (Table 2a; Fig 1B)

It was observed that the upregulated expression of four miRNAs was found to be increased with the increasing histopathological grade and clinical stage but also with the younger age, premenopausal stage, early menarche, smoking, higher mitotic activity, higher ki67 expression and lymph node negativity of TNBC. It is intriguing that expression of specifically miR-21 was 2 fold higher in benign breast tumor fibroadenoma (FC = 2.01) as compared to normal adjacent tissue (Fig 1C). However, the relative expression level of miR-221 and miR-210 did not significantly alter in benign tumors but it did show an increased expression. In contrast, the expression of miR-195 and miR-145 in all subtypes of breast cancer showed a consistent and significant downregulated expression which changed inversely as a function of the severity of breast cancer (Table 2a). The fold change expression level of miR-195 and miR-145 is presented in Fig 1A which showed a significant decrease as a function of hormone receptor negativity/severity of TNBC (Table 2a; Fig 1B). The expression of miR-195 and miR-145 also decreased with increasing histopathological grade and clinical stage and all other risk factor associated with TNBC (Fig 1C and S1 Table). The expression of these two miRs in fibroadenoma was nearly equal to that of normal adjacent tissue (Fig 1C). Interestingly, Inspite of being a known tumor suppressor, Let-7a was highly expressed in TNBC tissue but it was downregulated in fibroadenoma.

### Higher miRNA expression in tumor tissue is comparable to paired serum from TNBC patients

In consistent with the aberrant expression of specific miRNAs in breast tumor tissues, the selected panel of six miRNAs showed similar trend of significantly altered expression pattern in corresponding serum samples of TNBC patients though at a lower level than that of tissues. This is also true for other subtypes (DNBC, SNBC and TPBC) of breast cancer. As observed in tumor tissues, the miR-21, miR-221, miR-210 and Let-7a were also found to be overexpressed in their paired sera (13.99; 14.97, 8.39 and 11.84 fold change, p < 0.001; Table 2b) as compared to that of normal sera, though at a lower level than those observed for tissue miRNA (Fig 2A). Similar pattern of differential miRNA expression was recorded upon comparison with TNBC group with DNBC, SNBC, TPBC, including benign fibroadenoma (see Table 2b; Fig 2C). The relative expression of these four serum miRNA also showed good correlation with all clinicopathological and demographic risk factors (Table 2b and S1 Table; Fig 2B and 2C). miR-21 was also marginally increased in benign fibroadenoma while miR-221, miR-210 and Let-7a were underexpressed. The expression of miR-195 and miR-145 was found to be down-regulated in paired serum samples of TNBC patients and the difference was found to be statistically significant (Table 2b; Fig 2B). These two miRNAs showed similar trend of significantly a decreased expression in TPBC, SNBC and DNBC (Table 2b; Fig 2B). These two miRs were also found to be underexpressed (0.37 and 0.52 fold change) in benign fibroadenoma when compared to that of normal sera (Fig 2C). Let-7a showed the same level of higher expression pattern in sera but was lower than that of tumor tissue. It was underexpressed in fibroadenoma.

### Correlations of miRNA expression pattern between paired tissue and serum of TNBC

**Fig 3:**
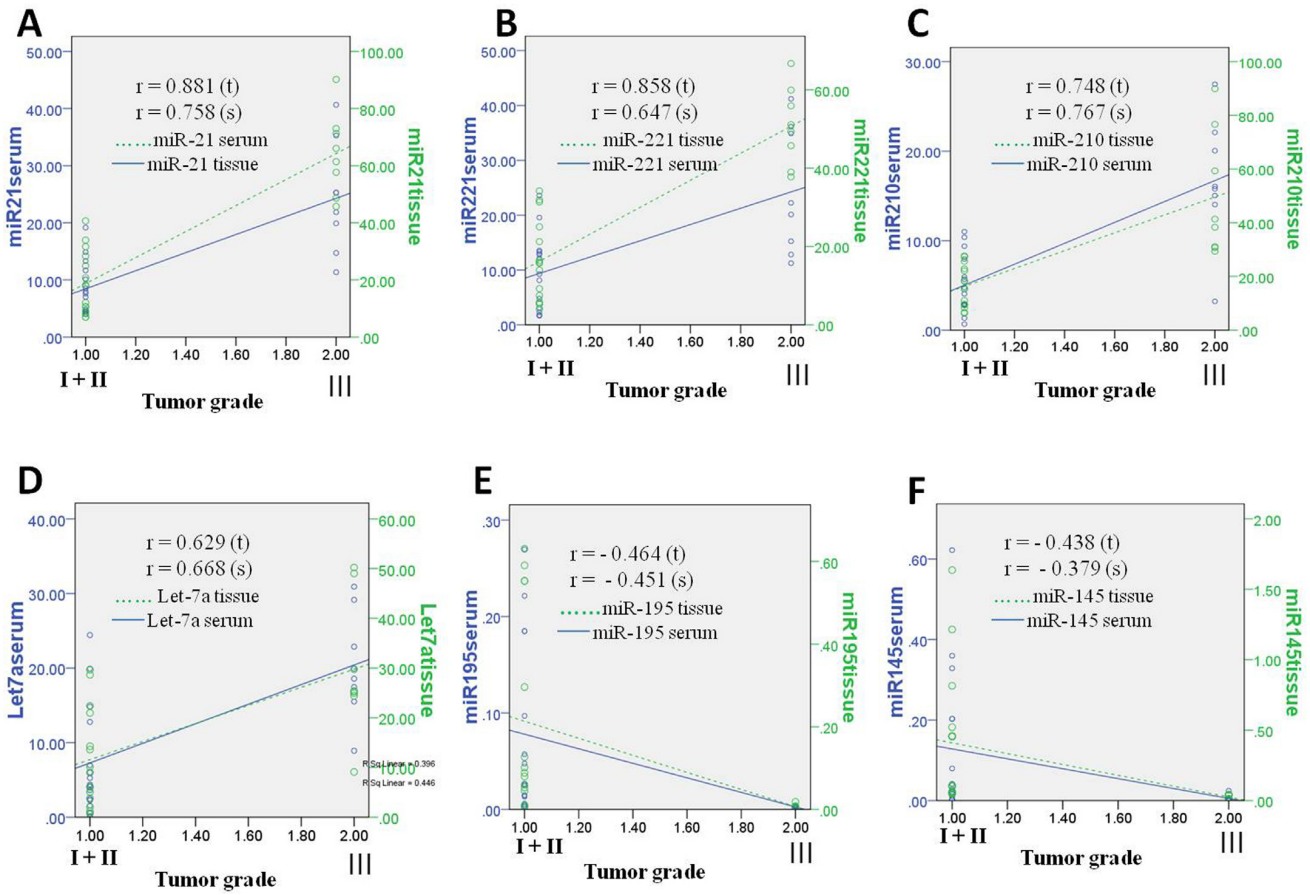
Pearson correlation coefficient of miRNAs in TNBC tissue and sera. Pearson correlation scatter plot of the fold-change expression level of miRNAs in tissue and sera from triple negative breast cancer patients which predicts a good correlation with tumor grade. (A) miR-21; (B) miR-221; (C) miR-210 and (D) Let-7a has showed significant positive correlation with respect to tumor grade. (E) miR-195; (F) miR-145 showed significant negative correlation or inverse correlation with increasing tumor grade.

To determine whether the serum miRNA expression could serve as a reliable noninvasive diagnostic or prognostic biomarker, the correlation of microRNA fold change in expression between paired tumor tissue and sera with increasing tumor grade or severity of triple-negative breast cancer was calculated using Pearson’s Correlation coefficient (r) (Fig 3). The results showed a significant correlation of the panel of five miRNAs, three over expressed miR-21, miR-221, miR210 and two downregulated miR-195 and miR-145 in the tissues (Pearson’s r = 0.881, r = 0.858, r = 0.748, r = −0.464 and r = −0.438 respectively, p<0.0001) and in their corresponding sera (r = 0.758, r = 0.647, r = 0.767, r = −0.451 and r = −0.379 respectively, p<0.0001). Statistically all were found highly significant (Fig 3A–3F). Despite being a tumor suppressor and in contrast to miR-195 and miR-145, miR Let-7a showed increased expression in TNBC tissue and sera and also showed a good correlation with tumor grades (r = 0.629 for tissue and r = 0.668 for sera, p<0.0001).

### Comparison of specific miRNA expression between paired tissue and sera of TNBC patients with that of breast cancer cell lines

We compared the expression profile of the six miRNAs observed in paired tissue and sera of different subtype of breast cancer with the respective breast cancer cell lines MCF-7 (ER+PR+EGFR-) and MDA-MB-231 (ER-PR-EGFR-) including normal breast epithelial cell lines MCF-10A to examine if results from fresh tumor tissue and serum can be comparable with that of the cell lines propagated for a long time in in-vitro culture (Fig 2D and Table 3). The results revealed a similar pattern of a statistically significant higher expression of four miRNAs in the TNBC cell line MDA-MB-231 followed by MCF-7 (DPBC) cells when compared with normal breast epithelial cell line, MCF10A and the results corroborated with that of corresponding fresh tumor tissue and paired sera. The expression of miR-195 and miR-145 also showed significant decreased expression in these two breast cancer cell lines. Taken together, the result demonstrated that four miRs i.e., miR-21, miR-221, miR-210 and Let-7a were significantly overexpressed in cell lines of TNBC as in TNBC tissue and sera. miR-21 exhibited the highest fold change expression in TNBC tissue than in sera and cell lines of TNBC (34.64 vs. 13.99 vs. 27.41 respectively). Interestingly, despite being a tumor suppressor, miR Let-7a showed increased expression in breast cancer cell lines.

**Table 3:** MicroRNA expression level in breast cancer cell lines.

	MCF-7		MDA-MB-231	
miRNA	FC±SEM	P value	FC±SEM	P value
**miR-21**	2.71±0.63	0.027*	27.41±3.37	0.001*
**miR-221**	8.42±1.15	0.0001*	21.91±1.53	0.001*
**miR-210**	12.42±1.21	0.0001*	24.08±3.41	0.0001*
**miR-195**	0.47±0.10	0.001*	0.03±0.01	0.0001*
**miR-145**	0.14±0.02	0.0001*	0.04±0.01	0.0001*
**Let-7a**	5.69±1.55	0.001*	7.55±2.39	0.001*

FC: Fold change; SEM: Standard Error Mean;

* Significant.

Next we compared the selected six miRNA expression with tumor grades and clinical stage. We observed that while miR-21, miR-221, miR-210 and Let-7a were overexpressed as a function of severity of lesion or tumor grade and stage of tissue and paired sera, miR-145 and miR-195 showed down regulation. The four upregulated miRNAs exhibited a very high expression at higher grade (III) malignancy.

### Specific miRNAs as potential diagnostic markers for TNBC as revealed by ROC curve

**Fig 4:**
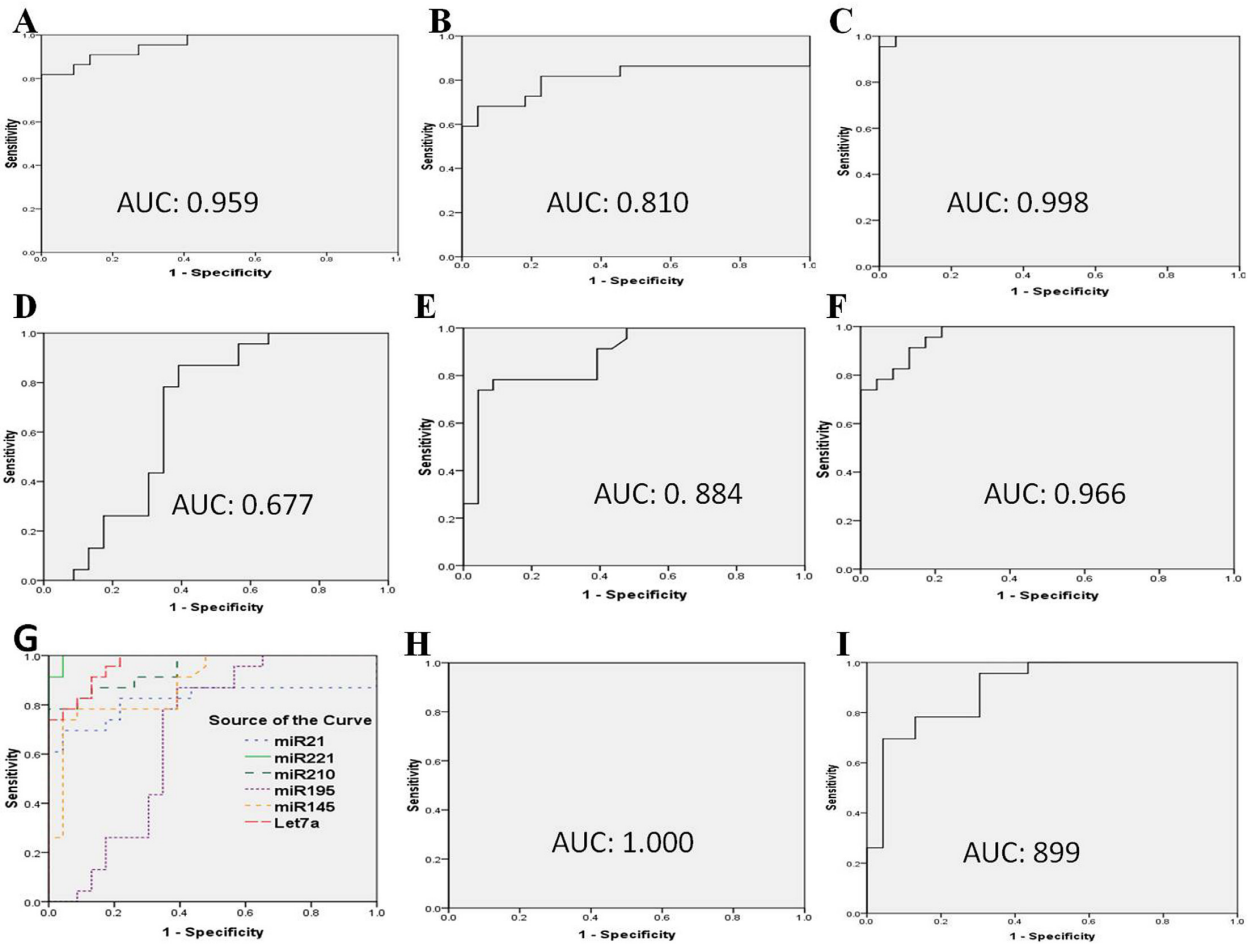
Receiver operating characteristic curve analysis of specific miRNAs in TNBC patients. Receiver operating characteristic curve of the diagnostic potential of the individual serum miRNAs. A (miR-21); B (miR-221); C (miR-210); D (miR-195); E (miR-145); F (Let-7a); (G) comparative ROC of all six miRNAs (miR-21; miR-221; miR-210; miR-195, miR-145 and Let-7a); (H) combined ROC of three miRs (miR-21; miR-221 and miR-210); (I) combined ROC of two downregulated miRNAs (miR-195 and miR-145). This predicts the diagnostic accuracy and expression level of miRNA signature that are able to discriminate between patients and healthy controls in the sera of triple negative breast cancer.

Of six miRNAs, the expression of four miRNAs, miR-21, miR-221, miR-210 and Let-7a were found to be significantly upregulated while the other two miRNAs, miR-195 and miR-145 were downregulated in both paired tumor tissue and sera of triple negative breast cancer patients. The diagnostic accuracy of six miRNA signatures to distinguish between triple negative breast cancer patients and healthy normal women was assessed by a ROC test (receiver operating characteristics curve; Table 4a and 4b and Fig 4).

**Table 4:** Receiver operating characteristic curve (ROC) analysis for the set of 5 microRNAs for differentiating TNBC in tissues and sera.

**(a) ROC for TNBC tissues (n = 23)**				
**miRNA**	**Sensitivity**	**Specificity**	**AUC (95% CI)**	**P value**
**miR-21**	100%	95%	0.9977 (0.9903–1.000)	p < 0.0001*
**miR-221**	100%	100%	1.000 (1.000–1.000)	p < 0.0001*
**miR-210**	95%	95%	0.9783 (0.9347–1.022)	p < 0.0001*
**miR-195**	73%	78%	0.7580 (0.6058–0.9103)	p = 0.0027*
**miR-145**	73%	60%	0.7013 (0.5489–0.8538)	p = 0.0193*
**Let-7a**	95%	95%	0.9773 (0.9411–1.014)	p < 0.0001*
**(b) ROC for TNBC sera (n = 23)**				
**miR-21**	95%	81%	0.9587 (0.9071–1.000)	p < 0.0001*
**miR-221**	81%	72%	0.8099 (0.6629–0.9570)	p = 0.0004*
**miR-210**	100%	100%	0.9979 (0.9911–1.000)	p < 0.0001*
**miR-195**	78%	65%	0.6767 (0.5098–0.8437)	p = 0.0400*
**miR-145**	78%	91%	0.8837 (0.7862–0.9812)	p < 0.0001*
**Let-7a**	91%	86%	0.9660 (0.9237–1.008)	p < 0.0001*

AUC: Area Under the Curve; CI: Confidence Interval;

* Significant.

ROC curve analysis performed to evaluate the diagnostic utility demonstrated that miR-21, miR-221, miR-210 and Let-7a were the most important biomarkers for discriminating not only TNBC but all other subtypes from healthy individuals. The miR-21 had a highest accuracy of area under the curve (AUC) of 0.9637 (95% confidence interval: 0.9394–0.9881, p < 0.0001) for tumor tissue as compared to other miRNAs and also highest for paired sera with AUC of 0.7864 (95% CI (Confidence Interval): 0.7127–0.8602, *p* < 0.0001), (Table 5a and 5b). The diagnostic sensitivity and specificity at optimal cut-off was 94% and 88% for identification of all subtypes of breast cancer indicating a very high diagnostic efficacy for tissue, 88% and 65% for sera. However, the downregulated miRNA showed lower AUC of 0.5078 (95% CI: 0.4201–0.5955, *p* < 0.0001) with a low sensitivity and specificity of 51% and 45% for tissue and for sera AUC was found to be 0.8043 (95% CI: 0.7369–0.8717, *p* < 0.0001) with sensitivity and specificity of 77% and 71% for miR-195 for all subtypes of breast cancer (Table 5a and 5b). The AUCs, confidence interval (CI), sensitivity and specificity with p value for each miRNA of all subtypes of breast cancer are presented in Table 5a and 5b. Similarly, the four highly upregulated miRNAs appeared to be the best classifiers for TNBC patients from healthy individuals with high accuracy AUC value of 0.998 (95% CI: 0.9903–1.000, *p* < 0.0001), 1.000 (95% CI: 1.000–1.000, p < 0.0001) and 0.978 (95% CI: 0.9347–1.000, p < 0.0001), 0.9773(95% CI: 0.9411–1.014, p < 0.0001) respectively for tissues (Table 4a and Fig 4A–4C). All the four upregulated miRs showed a higher sensitivity and specificity of 100% and 95% for miR-21, 100% and 100% for miR-221, 95% and 95% for miR-210 and 95% and 95% for Let-7a. For paired serum, miR-21 had a AUC value of 0.959 (95% CI: 0.9071–1.000, p < 0.0001) with 95% sensitivity and 81% specificity, miR-221 of 0.810 (95% CI: 0.6629–0.9570, p < 0.0001) with 81% sensitivity and 72% specificity, miR-210 of 0.998 (95% CI: 0.9911–1.000, p < 0.0001) with 100% sensitivity and 100% specificity and Let-7a of 0.966 (95% CI: 0.9237–1.008, p < 0.0001) with 91% sensitivity and 86% specificity (Table 4b).

**Table 5:** Receiver operating characteristic curve (ROC) analysis for the set of 6 microRNAs for total breast cancer tissues and sera.

**(a) ROC for total tumor tissue (n = 85)**				
**miRNA**	**Sensitivity**	**Specificity**	**AUC (95% CI)**	**P value**
**miR-21**	94%	88%	0.9637 (0.9394–0.9881)	p < 0.0001*
**miR-221**	88%	70%	0.8994 (0.8553–0.9436)	p < 0.0001*
**miR-210**	90%	71%	0.8602 (0.8027–0.9177)	p < 0.0001*
**miR-195**	51%	45%	0.5078 (0.4201–0.5955)	p = 0.05*
**miR-145**	60%	62%	0.5826 (0.4962–0.6691)	p = 0.0528*
**Let-7a**	80%	74%	0.8764(0.8235–0.9293)	p < 0.0001*
**(b) ROC for total cancer sera (n = 85)**				
**miR-21**	88%	65%	0.7864 (0.7127–0.8602)	p < 0.0001*
**miR-221**	65%	57%	0.6252 (0.5408–0.7096)	p = 0.0048*
**miR-210**	78%	61%	0.6369 (0.5497–0.7241)	p = 0.0020*
**miR-195**	77%	71%	0.8043 (0.7369–0.8717)	p < 0.0001*
**miR-145**	74%	56%	0.7339 (0.6575–0.8103)	p < 0.0001*
**Let-7a**	71%	67%	0.7620(0.6906–0.8335)	p < 0.0001

**Abbreviations:** AUC: Area under the curve; CI: confidence interval; ROC: receiver operating characteristic; miRNA: MicroRNA;

* Significant.

The AUC value, sensitivity and specificity of the two downregulated microRNAs, miR-195 and miR-145 are presented in Table 4a and 4b; Fig 4D and 4E. These findings together indicate immense potential of serum based miRNAs as reliable screening biomarkers for TNBC (see Table 4b).

When we combined the three upregulated miRNAs for the calculation of AUC value, it revealed a perfect AUC value of 1.0 (95% CI: 1.000–1.000), with a sensitivity and specificity of 100%. This appears to be the best diagnostic accuracy that could ever be achieved for the discrimination of TNBC patients from healthy individuals (Fig 4H). The combination of two downregulated miRNAs (miR-195 and miR-145) also revealed a good AUC of 0.899 (95% CI: 0.8082 to 0.9873) with an optimal sensitivity of 78% and specificity of 87% (Fig 4I). A screening approach employing any of these four significantly overexpressed miRNAs individually can still reveal a good but lower diagnostic accuracy when compared to the three upregulated miRNAs (miR-21; miR-221; miR-210) and/or two downregulated miRNAs (miR-195 and miR-145) combined together (Fig 4G–4I).

### Specific miRNA expression signature well correlates with clinicopathological characteristics of TNBC

The expression signatures of the panel of 6 selected miRNAs in paired tissue and sera have been correlated with the most common clinicopathological and demographic characteristics often associated as risk factors for the development of breast cancer (Table 1). The upregulated expression of four miRNAs, miR-21, miR-210, miR-221 and Let-7a showed a significant (p ≤ 0.05) correlation with several most common clinicopathological and demographic variables such as age, menopausal status, oral contraceptives use, Ki67 expression, tumor grade, clinical stage, and BMI of TNBC patients (Table 1 and S1 Table). Interestingly, instead of a higher lymphnode positivity, the majority of premenopausal TNBC patients were presented with lymphnode negative status (Table 1). The results revealed a significant correlation between increased expression of these four serum miRs (fold change 17.29; 10.60; 17.08 and 14.85 respectively) and younger age (<35 year) (69.56%), pre-menopausal status (73.91%), early age of menarche (<13 year), higher mitotic activity and increased Ki67 expression, increasing tumor grade and clinical stage and lymph node negativity of TNBC patients (S1 Table). Our study also showed a significantly (P< 0.05) higher expression of specifically miR-210, miR-221 (11.28; 18.87 fold change) in oral contraceptive users in TNBC. However, miR-21 (16.73 fold), inspite of being the most sensitive marker it did not show any significant correlation. A significant association (p<0.001) was also observed for an increased expression of miR-210 and miR-21 (11.41 and 18.81 fold change) with obesity and menarche (11.85 and 19.48 fold change) (see S1 Table). The over expression of miR-210 alone was found to be associated with BRCA1 mutation. However, expression of these four miRs either in tumor tissue or in sera did not show significant association with smoking, food habits, religion or BRCA1/ BRCA2 mutation in TNBC patients (S1 Table). The expression of two miRNAs (miR-195 and miR-145) was found to be significantly downregulated and when we correlated the expression of these two miRs with the above clinicopathological and demographic variables, we also found a good negative correlation. Thus the altered expression signature of all the six miRNAs changed as a function of hormone receptor negativity, clinicopathological variables and severity of TNBC patients and is reflected in both tissues and paired sera.

## Discussion

Early detection remains a mainstay of cancer screening particularly in the case of breast cancer. Since the current conventional screening tests are either hazardous or unreliable because of false positive (~10%) results, there is a need for identification of novel non-invasive or minimally invasive biomarkers for reliable and early detection of breast cancer. Body fluid based microRNA signature in blood plasma, serum and even urine offers an excellent non-invasive diagnostic tool for early detection of breast cancer [18–20]. miRNAs involved in various pathophysiological processes including control of gene expression and cell signalling have been considered to be potential tissue or body fluid based biomarkers for diagnosis, prognosis, therapeutic targets and classification of various cancers including breast cancer [18, 20–23].

We selected a panel of six specific miRNAs which function as oncogenes or tumor suppressors and they have multiple targets to play a pivotal role in breast carcinogenesis. miR-21 inhibits the expression of the tumor suppressor PDCD4 (programmed cell death-4) and PTEN (phosphatase and tensin homolog) gene [24, 25]. Similarly miR-221 targets the cell cycle inhibitor p27^Kip1^ and functions as an oncogene in breast cancer [26]. miR-221 inhibits ERα translation by direct interaction with the 3′-UTR of ERα and thus is responsible for ERα regulation at the post-transcriptional level and highly expressed in ERα negative breast cancer [27, 28]. The target genes of miRNA-210 are involved in cell proliferation, mitochondrial metabolism, DNA repair (RAD52), chromatin remodeling, and cell migration. The hsa-miR-210 overexpression is induced by hypoxia in a HIF-1alpha (Hypoxia inducing factor- 1α) and VHL (von Hippel-Lindau)-dependent fashion [29]. Johnson et al., [30] demonstrated that the expression of let-7 inversely correlates with expression of RAS protein. Also, several target genes of miR-145, such as c-Myc and MMP-11 (matrix metallopeptidase 11) have been identified and its critical role as a tumor suppressor in the p53 regulatory network has been demonstrated [31]. The level of miR-195 expression is inversely correlated with tumorigenesis and Raf-1 (rapidly accelerated fibrosarcoma) is a direct target of miR-195 and Raf-1 is overexpressed in many cancers including breast cancer [32].

Triple negative breast cancer (TNBC) exhibits a highly aggressive tumor phenotype and a worse prognosis compared to other breast cancer subtypes [33, 34]. There are only about 30% of women with metastatic TNBC with 5 years survival, and majority of patients eventually die of their disease. This is mainly due to high heterogeneity of the tumors and lack of definitive clinical determinants or TNBC-specific diagnostic and therapeutic targets. Therefore identification of specific molecular marker(s) for TNBC, preferably a non-invasive one is of immense importance for easy and reliable diagnosis of the disease early and to identify therapeutic target(s) to improve treatment outcomes [35–37]. The present study represents a large collection of paired serum and tumor samples with all clinical, epidemiological and demographic details of the patients including hormonal status and histopathological diagnosis that allowed us to do a meaningful correlation of microRNA signatures for early identification of TNBC. In this study, we report for the first time an exceptionally higher (~ 74%) prevalence of TNBC specifically in premenopausal women below the age of 35 years. A panel of six miRNAs, 3 oncogenic and 3 tumor suppressor miRNAs have been identified as possible markers in blood serum which represents the largest profile of human proteome and corresponds to tumor for an early and minimally invasive detection of TNBC. We find that three oncogenic miRNAs, miR-21, miR-221 and miR-210 were consistently overexpressed while two tumor suppressor miRNAs, miR-195 and miR-145 were always downregulated in paired tumor tissue and sera as well as cell lines of TNBC compared to TPBC or other subtypes of breast cancer. Most intriguing was the finding that one of the tumor suppressor miRNA, miR Let-7a showed consistently an increased expression in TNBC and other breast cancer subtypes and cell lines. The miRNA expression pattern well correlated with various clinicopathological and demographic risk factors and the panel appears to be a very attractive biomarker for clinical application. Although the fold change expression of 4 upregulated miRs (includes Let-7a) in TNBC tissue was significantly much higher (34.64, 28.35, 27.82, and 17.90 respectively) as compared to those in paired sera (13.99, 14.97, 8.39 and 11.84 respectively), the fold change expression in the sera samples was still significantly higher than that of adjacent normal tissue, normal sera or cell line (MCF-10A) (see Table 2a and 2b; Figs 1B and 2B). It is also intriguing that the fold changes in TPBC and SNBC were found to be insignificant when compared to that of adjacent controls. This needs further investigation.

A series of circulating microRNAs in blood have been identified as biomarkers by several authors [18, 19, 21, 22, 33, 38–41] but there are wide variations in results due to use of a variety of biological samples such as plasma, serum, whole blood or even urine [21, 42–45] and use of various experimental approaches such as next generation sequencing, RT-PCR, and targeted analysis of specific microRNAs [35, 46]. Surprisingly enough, a recent report from India by Mishra et al.,[44] could not find any significant correlation in miRNA expression profile between PBMC, plasma and tumor tissues of breast cancer patients when compared to controls. They suggest that this is perhaps because of selective secretion of specific miRNAs in peripheral blood when compared to that in plasma or tumor tissue. However, Chan et al.,[17] observed seven common miRNAs that are overexpressed in both breast tumours and sera from breast cancer patients, and one microRNA that was down-regulated. Earlier in several studies, differential expressions of circulating miRNAs have also been proved to be potent biomarkers for breast cancer diagnosis and prognosis [15, 23, 41]. Interestingly, several authors identified Let-7a as a downregulated miRNA in breast cancer [47–49]. Although several reports indicate an alteration in expression of miR-21, miR-145, miR-221, miR-195 and Let-7a in a variety of breast cancer types [16, 50–54] but none is specific to TNBC. We find a highly significant overexpression of Let-7a in TNBC with a fold change value of 17.90 in tissue, 11.84 in sera and 7.55 fold change in cell line (Tables 2a, 2b and 3). It is suggested that the level of expression of miRNA depends not only on the type but also on the stage and grade of tumors. Radojicic et al.,[16] demonstrated overexpression of miR-21, miR-210 and miR-221 while miR-10b, miR-145, miR-205, miR-122a were significantly downregulated in TNBC. Hu et al.,[55] found miR-93 expression in TNBC tissues was significantly higher than that in non-TNBCs. It indicates that expression of miRNA changes not only between subtypes, stages and grades of breast of cancer but also between different ethnic or geographic population. Till date no specific miRNA biomarker has been reported for TNBC patients from India where TNBC is highly (~74%) prevalent in less than 35 year premenopausal women.

Along with TNBC, we have also evaluated the expression profile of these six miRNAs in DNBC (ER-/PR-/Her2neu+), SNBC (ER-/PR+/Her2neu+) and TPBC (ER+/PR+/Her2neu+) patients including benign fibroadenoma and healthy subjects as controls. Our study demonstrates that serum miRNAs can distinguish not only the patients with TNBC from healthy controls but also from TPBC, SNBC, DNBC and benign fibroadenoma patients. Curiously, benign fibroadenoma was found to have slightly higher miRNA expression than that of adjacent normal controls but certainly much lesser than those in BC patients. This increase is possibly due to increased growth and proliferative potential of fibroadenoma tissues (Fig 1C and 1F). Therefore, fibroadenoma tissue samples certainly cannot be used as a normal control. A highly significant correlation has been observed for aberrant tissue overexpression of miR-21, miR-221, miR-210 and Let-7a between tumor grade and stage of TNBCs and their corresponding sera.

Further, Pearson’s correlation coefficient analysis of 4 upregulated miRs (miR-21, miR-221, miR-210 and Let-7a) demonstrated a highly significant positive correlation of miRNA expression profiles between paired sera (r = 0.758, r = 0.647, r = 0.767 and r = 0.668 respectively, p<0.0001) and tissue samples (r = 0.881, r = 0.858, r = 0.748 and r = 0.629 respectively, p<0.0001) and it increased with the increasing severity (grade/ stage) of breast cancer (Fig 3A–3D). In contrast, the two downregulated miRs (miR-195 and miR-145) showed inverse correlation, (r = −0.464 and r = −0.438, p < 0.0001) for tissue and (r = −0.451 and r = −0.379, p<0.0001) for sera as their expression decreased with the severity of the disease (Fig 3E and 3F). Thus this blood-based panel of six specific miRNAs appears to be a useful biomarker for detecting and/or screening of TNBC. All of the six analyzed miRNAs showed comparable absolute level in both paired sera and tissue of TNBC and other breast cancer subtypes and in corresponding established breast cancer cell lines.

The aberrant over expression of the four miRNAs and consistent down regulation of two miRs showed a good correlation not only with tumor grade, clinical stage, lymph node status and hormone receptor expression but also with majority of clinicoepidemiological and demographic risk factors often associated with the development of breast cancer (see S1 Table and Table 2a and 2b). It is most alarming and of immense public health concern that as high as 74% of TNBC in India is prevalent specifically in premenopausal stage with an age as low as 35 years which is the most productive and prime time of reproductive and social life of women. Additionally, majority of known risk factors such as early age menarche, smoking, high mitotic activity, over expression of ki67 and lymph node positivity are significantly higher in TNBC patients (Table 1). Though oral contraceptive use, food habits, religion and BMI played an important role, but they failed to show significant association. So far there is so far no report from any part of the globe that shows such a high prevalence of TNBC in premenopausal (>35) women. There are several reports [56–60], which show prevalence of TNBC that varies from 29% to 52% only in the premenopausal age between 40 to 45 years but there is one report from Morocco that reported prevalence of only about 18% TNBC below the age of 35 years. We observed as high as 69.56% of TNBCs are smokers and 73.91% had early age menarche which is also associated with a higher BMI. We also observed a higher (78.26%) mitotic activity and positivity for ki67 (78.26%) in TNBC.

In this study, we employed ROC curve analysis to demonstrate the diagnostic utility of six miRNAs derived from sera which were able to distinguish between TNBC and healthy individuals (Table 4a and 4b and Fig 4A–4I). The sensitivity and specificity of each of the six miRNAs were analyzed at optimal cut-off for TNBC tissue and sera are found to be highly significant (Table 4a and 4b). Furthermore, a significant improvement in the diagnostic potential and accuracy was achieved when ROC analysis of three overexpressed miRNAs were combined. For this miRNA panel we were able to reach a discriminatory power of AUC = 1.000, with 100% sensitivity and 100% specificity. Even scoring with the two downregulated miRNAs (miR-195 and miR-145), the accuracy was improved with an AUC of 0.899, and 78% sensitivity and 87% specificity. Outcomes from these statistical analyses showed that the three upregulated miRNAs in combination could be a potential molecular signature with 100% efficacy for early diagnosis of TNBC (Fig 4H and 4I). Owing to a non-invasive nature of serum, together with its higher sensitivity and specificity, the 6-serum based miRNA panel offers a new perspective in the detection of TNBC at an early stage. Areas under the curve (AUC) and p-values were highly significant for miR-21, miR-221, miR-210 and Let-7a in both tumor tissue and sera suggesting for an enormous potential of these four upregulated miRNAs as biomarker for TNBC. Use of these miRs along with two downregulated miRs showed an excellent ability to discriminate between TNBC patients and other BC subtypes and healthy individuals. Even if three upregulated miRs are used as a single marker, it can effectively classify 95% of the TNBC samples.

It is important that we could identify a panel of six specific serum based microRNAs that show excellent correlation with corresponding tumor tissues and cell lines and also with the majority of well-established clinicopathological and demographic risk factors associated with TNBC thus making it a reliable minimally invasive biomarker for diagnosis and prognosis of TNBC. This will also have immense importance in guiding breast cancer treatment and management. Since the use of whole blood will lead to isolation of microRNAs from many different cell types including those within the blood cells, and not just circulating microRNAs in plasma or serum, there is a need for utmost caution when comparing microRNA profiles derived from these sources. Although serum and plasma are considered equivalent, concentration of microRNA is often higher in serum [17, 43]. The possibility of a serological test that can augment histological information of a tumour without the need for biopsy is an exciting approach for research and clinical application. Serum-derived miRNAs are stably present and the results are reproducible among individuals.

In conclusion, it is suggested that the serum-based six specific microRNAs presented here can reliably be employed for noninvasive and differential diagnosis and/or prognosis of TNBCs especially the early stage TNBCs in comparison to TPBCs or other subtypes of breast cancer. Additional validation studies may be required to prove the discriminative ability of these miRNAs either singly or in combination for a reliable diagnosis of triple negative breast cancer.

## Supporting Information

S1 Table The expression level of miRNAs in relation to Clinicopathological characteristics of triple negative breast cancer tissue and paired sera. (DOCX)

S2 Table The Original raw data of microRNA expression including clinical characteristics. (XLSX)

## Abbreviations

miR: microRNA
TNBC: triple negative breast cancer
DNBC: double negative breast cancer
SNBC: Single negative breast cancer
AUC: area under curve
ROC: receiver operating characteristics
CI: confidence interval
ER: estrogen receptors
PR: progesterone receptor
HER2: human epidermal growth factor receptor 2
