# Supplementary material for: Identification of Specific miRNA Signature in Paired Sera and Tissue Samples of Indian Women with Triple Negative Breast Cancer
Source: PLoS One. 2016 Jul 12;11(7):e0158946. doi: 10.1371/journal.pone.0158946 (PMC4942139; doi:10.1371/journal.pone.0158946)
Supplement: S1 Table — (DOCX) [file pone.0158946.s001.docx]

**S1Table. The expression level of miRNAs in relation to Clinicopathological characteristics of triple negative breast cancer tissue and paired sera.**

| **S. NO.** | **Clinico-**  **pathological**  **variables** | **Category** | **Number of**  **cases n (%)** | **miR-210**  **Mean±SE** | **P value** | **miR-221**  **Mean±SE** | **P value** | **miR-21**  **Mean±SE** | **P value** | **miR-195**  **Mean±SE** | **P value** | **miR-145**  **Mean±SE** | **P value** | **Let-7a**  **Mean±SE** | **P value** | **miR-145**  **Mean±SE** |  | **P value** |  |
| --- | --- | --- | --- | --- | --- | --- | --- | --- | --- | --- | --- | --- | --- | --- | --- | --- | --- | --- | --- |
| **1.** | **Age Range**  **(Mean ±SD)** | (21-70) | \| 23(36.52±10.27) \|  \| \| --- \| --- \| | 27.83±4.52(t)  8.39±1.34(s) |  | 28.36±4.04(t)  14.97±2.24(s) |  | 34.65±5.22(t)  14.00±2.11(s) |  | 0.14±0.04 (t)  0.05±0.01(s) |  | 0.27±0.08 (t)  0.08±0.03(s) |  | 17.90±2.93 (t)  11.84±1.99 (s) |  |  |  |  |  |
| **2.** | **Age Group**  **distribution** | <35 | 16(69.56) | 35.43±5.45(t)  10.60±1.56(s) | **0.008***  **0.010*** | 34.37±4.58(t)  17.08±2.51(s) | **0.021***  0.160 | 41.14±5.85(t)  17.29±2.62(s) | **0.058***  **0.015*** | 0.06±0.03(t)  0.02±0.01(s) | **0.010***  **0.005*** | 0.09±0.03(t)  0.03±0.02(s) | **0.001***  **0.041*** | 22.18±3.58(t)  14.85±2.45(s) | **0.024***  **0.019*** |  |  |  |  |
|  |  | ≥35 | 7(30.43) | 10.43±2.16(t)  3.35±1.29(s) |  | 14.61±5.60(t)  10.16±4.35(s) |  | 19.81±8.95(t)  6.468±0.95(s) |  | 0.30±0.09(t)  0.13±0.04(s) |  | 0.68±0.21(t)  0.17±0.08(s) |  | 8.12±2.68(t)  4.96±1.53(s) |  |  |  |  |  |
| **3.** | **Menopausal**  **Status** | Pre-  menopausal | 17(73.91) | 34.69±5.17(t)  10.59±1.47(s) | **0.007***  **0.003*** | 35.03±4.36(t)  18.12±2.58(s) | **0.003***  **0.014*** | 43.01±5.81(t)  16.93±2.48(s) | **0.004***  **0.01*** | 0.06±0.03(t)  0.02±0.01(s) | **0.002***  **0.001*** | 0.09±0.03(t)  0.03±0.02(s) | **0.0001***  **0.005*** | 22.18±3.37(t)  14.73±2.30(s) | **0.010***  **0.011*** |  |  |  |  |
|  |  | Post-  menopausal | 6(26.08) | 8.36±0.73(t)  2.18±0.65(s) |  | 9.42±2.51(t)  6.03±1.65(s) |  | 10.94±1.51(t)  5.65±0.59(s) |  | 0.36±0.09(t)  0.15±0.04(s) |  | 0.78±0.56(t)  0.23±0.09(s) |  | 5.74±1.46(t)  3.66±0.96(s) |  |  |  |  |  |
| **4.** | **Menarche** | <13 | 17(73.91) | 40.17±6.00(t)  11.85±1.75(s) | **0.002***  **0.003*** | 30.89±5.19(t)  15.39±2.90(s) | 0.301  0.760 | 50.16±6.31(t)  19.48±2.88(s) | **0.0001***  **0.001*** | 0.16±0.06(t)  0.06±0.02(s) | 0.469  0.447 | 0.18±0.11(t)  0.03±0.02(s) | 0.220  **0.005*** | 23.69±3.75(t)  16.07±2.45(s) | **0.010***  **0.005*** |  |  |  |  |
|  |  | ≥13 | 6(26.08) | 11.77±1.48(t)  3.90±0.94(s) |  | 21.15±4.08(t)  13.77±2.79(s) |  | 14.47±2.09(t)  6.85±0.83(s) |  | 0.08±0.01(t)  0.03±0.01(s) |  | 0.41±0.13(t)  0.23±0.09(s) |  | 8.88±2.85(t)  5.25±1.96(s) |  |  |  |  |  |
| **5.** | **Oral**  **contraceptives** | Ever | 15(65.21) | 36.44±5.70(t)  11.28±1.56(s) | **0.006***  **0.001*** | 36.95±4.71(t)  18.87±2.88(s) | **0.002***  **0.013*** | 42.76±6.41(t)  16.73±2.74(s) | **0.030***  0.070 | 0.06±0.04(t)  0.02±0.01(s) | **0.015***  **0.001*** | 0.10±0.41(t)  0.03±0.02(s) | **0.004***  **0.005*** | 21.84±3.80(t)  14.37±2.59(s) | **0.064***  0.082 |  |  |  |  |
|  |  | Never | 8(34.78) | 11.67±2.51(t)  2.98±0.85(s) |  | 12.24±2.73(t)  7.65±1.61(s) |  | 19.42±6.46(t)  8.85±2.49(s) |  | 0.28±0.08(t)  0.15±0.04(s) |  | 0.60±0.20(t)  0.23±0.09(s) |  | 10.51±3.37(t)  7.08±2.39(s) |  |  |  |  |  |
| **6.** | **Smoking**  **Status** | Yes | 16(69.56) | 30.31±6.42(t)  9.55±1.82(s) | 0.419  0.202 | 31.39±5.37(t)  16.55±3.00(s) | 0.266  0.298 | 38.34±6.82(t)  15.34±2.77(s) | 0.295  0.346 | 0.69±0.53(t)  0.06±0.02(s) | 0.405  0.623 | 0.31±0.12(t)  0.09±0.04(s) | 0.504  0.790 | 18.48±3.92(t)  12.38±2.50(s) | 0.772  0.692 |  |  |  |  |
|  |  | No | 7(30.43) | 22.14±1.84(t)  5.76±1.11(s) |  | 21.42±4.48(t)  11.36±2.44(s) |  | 26.18±6.74(t)  10.91±2.73(s) |  | 0.10±0.06(t)  0.04±0.02(s) |  | 0.18±0.07(t)  0.07±0.05(s) |  | 16.57±3.92(t)  10.60±3.41(s) |  |  |  |  |  |
| **7.** | **Food**  **habits** | Vegetarian | 9(39.13) | 35.10±8.48(t)  9.71±2.22(s) | 0.204  0.445 | 30.58±6.77(t)  18.93±5.00(s) | 0.669  0.162 | 40.84±9.63(t)  16.30±9.95(s) | 0.354  0.393 | 0.12±0.07(t)  0.03±0.02(s) | 0.762  0.499 | 0.23±0.13(t)  0.11±0.07(s) | 0.706  0.488 | 19.13±4.88(t)  11.69±3.13(s) | 0.745  0.954 |  |  |  |  |
|  |  | Non- Vegetarian | 14(60.86) | 23.14±4.92(t)  7.55±1.71(s) |  | 26.92±5.18(t)  12.42±1.67(s) |  | 30.66±6.00(t)  12.50±10.33(s) |  | 0.15±0.06(t)  0.06±0.02(s) |  | 0.30±0.12(t)  0.06±0.02(s) |  | 17.10±3.78(t)  11.94±2.68(s) |  |  |  |  |  |
| **8.** | **Religion**  **Status** | Hindu | 10(43.47) | 35.10±8.48(t)  9.54±1.99(s) | 0.243  0.470 | 29.16±6.22(t)  18.25±4.52(s) | 0.865  0.206 | 39.59±8.70(t)  15.84±3.00(s) | 0.419  0.456 | 0.12±0.06(t)  0.04±0.01(s) | 0.745  0.511 | 0.21±0.12(t)  0.10±0.06(s) | 0.589  0.607 | 20.08±4.47(t)  12.51±2.92(s) | 0.527  0.776 |  |  |  |  |
|  |  | Muslim | 13(56.52) | 23.72±5.27(t)  7.52±1.85(s) |  | 27.73±5.52(t)  12.44±1.81(s) |  | 30.84±6.48(t)  12.57±2.98(s) |  | 0.15±0.06(t)  0.06±0.02(s) |  | 0.31±0.13(t)  0.07±0.02(s) |  | 16.22±3.97(t)  11.32±2.81(s) |  |  |  |  |  |
| **9.** | **Mitotic**  **activity**  **Status** | High | 18(78.26) | 33.38±5.05(t)  10.26±1.42(s) | **0.016***  **0.005*** | 33.38±4.43(t)  17.38±2.54(s) | **0.014***  **0.038*** | 41.07±5.81(t)  16.25±2.44(s) | **0.016***  **0.04*** | 0.06±0.03(t)  0.02±0.01(s) | **0.0001***  **0.0001*** | 0.13±0.05(t)  0.04±0.02(s) | **0.001***  **0.036*** | 21.51±3.24(t)  14.29±2.21(s) | **0.015***  **0.016*** |  |  |  |  |
|  |  | Low | 5(21.73) | 7.82±0.61(t)  1.66±0.48(s) |  | 10.26±2.90(t)  6.28±2.00(s) |  | 11.50±1.72(t)  5.87±0.68(s) |  | 0.41±0.09(t)  0.17±0.04(s) |  | 0.77±0.28(t)  0.21±0.10(s) |  | 4.87±1.43(t)  3.00±0.86(s) |  |  |  |  |  |
| **10.** | **Ki67**  **(cell**  **proliferation**  **marker)** | Positive | 18(78.26) | 33.38±5.05(t)  10.26±1.42(s) | **0.016***  **0.005*** | 33.38±4.43(t)  17.38±2.54(s) | **0.014***  **0.038*** | 41.07±5.81(t)  16.25±2.44(s) | **0.016***  **0.04*** | 0.06±0.03(t)  0.02±0.01(s) | **0.0001***  **0.0001*** | 0.13±0.05(t)  0.04±0.02(s) | **0.001***  **0.036*** | 21.51±3.24(t)  14.29±2.21(s) | **0.015***  **0.016*** |  |  |  |  |
|  |  | Negative | 5(21.73) | 7.82±0.61(t)  1.66±0.48(s) |  | 10.26±2.90(t)  6.28±2.00(s) |  | 11.50±1.72(t)  5.87±0.68(s) |  | 0.41±0.09(t)  0.17±0.04(s) |  | 0.77±0.28(t)  0.21±0.10(s) |  | 4.87±1.43(t)  3.00±0.86(s) |  |  |  |  |  |
| **11.** | **Histo-**  **pathological**  **grade** | I+II | 15(65.21) | 16.22±1.97(t)  5.18±0.89(s) | **0.0001***  **0.0001*** | 18.94±3.45(t)  9.9134±1.81(s) | **0.0001***  **0.023*** | 22.84±5.08(t)  8.76±1.22(s) | **0.001***  **0.0001*** | 0.21±0.06(t)  0.07±0.02(s) | **0.026***  **0.034*** | 0.41±0.12(t)  0.12±0.04(s) | **0.036***  0.075 | 11.57±2.50(t)  7.27±1.89(s) | **0.001***  **0.0001*** |  |  |  |  |
|  |  | III | 8(34.78) | 48.19±9.31(t)  16.71±2.49(s) |  | 46.00±5.89(t)  24.23±4.10(s) |  | 56.76±6.52(t)  24.28±3.46(s) |  | 0.01±0.002(t)  0.003±0.001(s) |  | 0.02±0.01(t)  0.01±0.003(s) |  | 29.75±4.83(t)  20.40±2.53(s) |  |  |  |  |  |
| **12.** | **Clinical**  **Staging** | Stage I+II | 12(52.17) | 14.22±2.06(t)  4.08±0.80(s) | **0.0001***  **0.0001*** | 14.53±2.90(t)  9.26±1.97(s) | **0.0001***  **0.005*** | 14.72±2.05(t)  7.36±0.97(s) | **0.0001***  **0.001*** | 0.26±0.07(t)  0.10±0.02(s) | **0.002***  **0.004*** | 0.49±0.14(t)  0.15±0.05(s) | **0.007***  **0.015*** | 8.38±2.25(t)  4.76±1.50(s) | **0.0001***  **0.0001*** |  |  |  |  |
|  |  | Stage III+IV | 11(47.82 ) | 42.66±6.85(t)  13.10±1.82(s) |  | 43.43±4.63(t)  21.19±3.32(s) |  | 56.38±5.47(t)  20.07±3.05(s) |  | 0.01±0.001(t)  0.003±0.001(s) |  | 0.03±0.01(t)  0.004±0.002(s) |  | 28.28±3.58(t)  19.57±2.04(s) |  |  |  |  |  |
| **13.** | **Lymph**  **node**  **status** | Positive | 7(30.43) | 52.47±8.84(t)  14.88±2.27(s) | **0.001***  **0.001*** | 45.53±6.78(t)  22.70±4.38(s) | **0.003***  **0.019*** | 59.07±7.04(t)  25.26±3.53(s) | **0.001***  **0.001*** | 0.01±0.002(t)  0.003±0.001(s) | **0.047***  0.062* | 0.02±0.01(t)  0.01±0.003(s) | 0.063  0.112 | 30.52±5.50(t)  21.10±2.81(s) | **0.002***  **0.001*** |  |  |  |  |
|  |  | Negative | 16(69.56) | 17.04±2.02(t)  5.56±1.07(s) |  | 20.84±3.74(t)  11.58±2.17(s) |  | 23.96±4.88(t)  9.06±1.38(s) |  | 0.20±0.05(t)  0.07±0.02(s) |  | 0.38±0.11(t)  0.12±0.04(s) |  | 12.37±2.48(t)  7.79±1.84(s) |  |  |  |  |  |
| **14.** | **BMI (kg/m^2^)** | Obesity  ( ≥34) | 14(60.86) | 38.61±5.77(t)  11.41±1.67(s) | **0.001***  **0.003*** | 36.65±5.12(t)  18.06±3.10(s) | **0.007***  0.08 | 47.86±6.28(t)  18.81±2.75(s) | **0.001***  **0.002*** | 0.05±0.04(t)  0.01±0.01(s) | **0.017***  **0.008*** | 0.07±0.03(t)  0.03±0.02(s) | **0.002***  **0.040*** | 23.82±3.69(t)  16.14±2.42(s) | **0.008***  **0.009*** |  |  |  |  |
|  |  | Non-obesity (<25) | 9(39.13) | 11.05±1.45(t)  3.70±1.03(s) |  | 15.44±3.78(t)  10.16±2.52(s) |  | 14.09±2.29(t)  6.50±0.83(s) |  | 0.27±0.07(t)  0.11±0.03(s) |  | 0.59±0.18(t)  0.16±0.06(s) |  | 8.68±2.90(t)  5.78±2.13(s) |  |  |  |  |  |
| **15.** | **Hereditary**  **status** | Hereditary | 2 (8.6) | 21.61±6.05(t)  5.37±2.56(s) | 0.682  0.501 | 20.70±4.31(t)  12.53±0.36(s) | 0.571  0.746 | 44.86±16.52(t)  18.47±6.80(s) | 0.558  0.526 | 0.06±0.05(t)  0.02±0.02(s) | 0.622  0.675 | 0.07±0.02(t)  0.01±0.01(s) | 0.502  0.502 | 24.80±3.83(t)  17.36±2.53(s) | 0.068  0.406 |  |  |  |  |
|  |  | Sporadic | 21 (91.30) | 28.41±4.93(t)  8.68±1.45(s) |  | 29.08±4.39(t)  15.20±2.45(s) |  | 33.67±5.57(t)  13.56±2.24(s) |  | 0.14±0.04(t)  0.05±0.02(s) |  | 0.29±0.09(t)  0.09±0.03(s) |  | 11.42±2.32(t)  11.31±2.14(s) |  |  |  |  |  |
| **16.** | **BRCA1**  **Status** | Mutated | 13(56.52) | 37.42±6.61(t)  11.74±1.71(s) | **0.012***  **0.002*** | 36.15±5.40(t)  18.17±3.03(s) | **0.024***  0.105 | 39.72±7.00(t)  17.21±3.08(s) | 0.278  0.082 | 0.08±0.04(t)  0.02±0.01(s) | 0.138  0.083 | 0.10±0.04(t)  0.04±0.02(s) | **0.029***  0.143 | 22.70±4.28(t)  14.57±2.90(s) | 0.060  0.121 |  |  |  |  |
|  |  | Non-mutated | 10(43.47) | 15.34±2.97(t)  4.05±1.17(s) |  | 18.22±4.58(t)  10.80±2.99(s) |  | 28.04±7.74(t)  9.80±2.26(s) |  | 0.21±0.08(t)  0.09±0.03(s) |  | 0.49±0.17(t)  0.14±0.06(s) |  | 11.66±2.99(t)  8.29±2.30(s) |  |  |  |  |  |
| **17.** | **BRCA2**  **Status** | Mutated | 4(17.39) | 40.10±17.82(t)  9.14±336(s) | 0.221  0.805 | 33.09±16.44(t)  13.23±8.04(s) | 0.603  0.732 | 30.25±12.64(t)  15.49±6.94(s) | 0.709  0.753 | 0.23±0.14(t)  0.07±0.04(s) | 0.358  0.697 | 0.44±0.28(t)  0.24±0.15(s) | 0.394  **0.021*** | 28.31±12.32(t)  16.59±7.76(s) | 0.104  0.285 |  |  |  |  |
|  |  | Non-mutated | 19(82.60) | 25.24±4.13(t)  8.24±1.50(s) |  | 27.36±3.79(t)  15.33±2.26(s) |  | 35.57±5.87(t)  13.67±2.20(s) |  | 0.12±0.04(t)  0.05±0.02(s) |  | 0.24±0.09(t)  0.05±0.02(s) |  | 15.70±2.42(t)  10.84±1.85(s) |  |  |  |  |  |

**Abbreviations:** (t): Tissue; (s): Serum; S.E: Standard Error**;** * Significant
